# Microbial transfers from permanent grassland ecosystems to milk in dairy farms in the Comté cheese area

**DOI:** 10.1038/s41598-021-97373-6

**Published:** 2021-09-13

**Authors:** N. Chemidlin Prévost-Bouré, B. Karimi, S. Sadet-Bourgeteau, C. Djemiel, M. Brie, J. Dumont, M. Campedelli, V. Nowak, P. Guyot, C. Letourneur, V. Manneville, F. Gillet, Y. Bouton

**Affiliations:** 1 UMR 1347 Agroécologie - AgroSup Dijon – INRAE - Université Bourgogne – Université Bourgogne Franche-Comté, 21000 Dijon, France; 2 AgroSup Dijon, 26 boulevard du Dr Petitjean, 21000 Dijon, France; 3 Comité Interprofessionnel de Gestion du Comté – Unité R&D, Bâtiment INRAE URTAL, 39800 Poligny, France; 4 Institut de l’Elevage, 63170 Aubière, France; 5 Université Bourgogne Franche-Comté, UMR6249 Chrono-Environnement, 25030 Besançon, France

**Keywords:** Agroecology, Community ecology, Microbial ecology, Agroecology, Community ecology, Microbial ecology

## Abstract

The specificity of dairy Protected Designation of Origin (PDO) products is related to their “terroir” of production. This relationship needs better understanding for efficient and sustainable productions preserving the agroecological equilibrium of agroecosystems, especially grasslands. Specificity of PDO Comté cheese was related to the diversity of natural raw milk bacterial communities, but their sources need to be determined. It is hypothesized that raw milk indigenous microbial communities may originate from permanent grazed grasslands by the intermediate of dairy cows according to the sequence soil–phyllosphere–teat–milk. This hypothesis was evaluated on a 44 dairy farms network across PDO Comté cheese area by characterizing prokaryotic and fungal communities of these compartments by metabarcoding analysis (16S rRNA gene: V3–V4 region, 18S rRNA gene: V7–V8 region). Strong and significant links were highlighted between the four compartments through a network analysis (0.34 < r < 0.58), and were modulated by soil pH, plant diversity and elevation; but also by farming practices: organic fertilization levels, cattle intensity and cow-teat care. This causal relationship suggests that microbial diversity of agroecosystems is a key player in relating a PDO product to its “terroir”; this under the dependency of farming practices. Altogether, this makes the “terroir” even more local and needs to be considered for production sustainability.

## Introduction

Today, agriculture is committed to a transition to sustainable development with the objective of minimizing the environmental impacts of agricultural practices together with positive economic margins and preserving product quality. In this context, grasslands need a particular focus. Indeed, grasslands host high levels of biodiversity either in terms of plant species^1,2^ or microbial taxa^3^. These high levels of biodiversity support key ecosystem services for agriculture, especially primary production^4–6^ and nutrients cycling^7–9^; but also the stability of grasslands ecosystems functions according to the levels of functional redundancy among taxa^10^. Their productivity and diversity also support many Protected Designation of Origin (PDO) dairy productions in France, recognized worldwide for their quality and specificity. This support is quantitative since animal feeding is mainly based on forage resources and qualitative since the diversity of permanent grasslands is closely related to final product specificity. For example, in the PDO Comté (France, Jura Mountains) or PDO Abundance (France, Alps Mountains), variations in cheese organoleptic characteristics have been related to the “terroir” of production by means of floristic composition of grasslands^11–14^ which is affected by climatic and soil conditions^14^. In addition, raw milk cheese specificity such as Comté cheese has been demonstrated to be directly related to the microbial communities naturally occurring in raw milk^15,16^. These microbial communities naturally occurring in raw milk represent a minor part of the milk microbial community (e.g.* mesophilic lactobacilli*) when starter is added to milk for cheese production, but they become more and more dominant during ripening of pressed-cooked cheeses and affect cheese sensory characteristics^17,18^. Several studies conducted in different geographic dairy production regions in France: Franche-Comté^19^, Massif Central^20^, and in Ireland, county of Cork^21^ showed that these natural microbial communities of raw milk mainly originated from cow-teats. This suggests that cow-teat would be a hub in determining raw-milk microbiota and therefore the specificity of raw milk products like PDO Comté cheese. Studies focusing on cow-teat microbiota have demonstrated that it was highly responsive to changes in the environment explored by cows^22,23^, suggesting that it would result of inputs from multiple environmental sources which need to be traced. The microbial phyla found on cow-teat (e.g.* Firmicutes*, *Actinobacteria*, *Proteobacteria*) are regularly observed in grassland ecosystems grazed by cows either in soil^3,24^ or phyllosphere^25^. This leads to the hypothesis that microbial communities naturally occurring in raw milk may originate from grassland ecosystems through microbial transfers along the sequence soil–phyllosphere–cow-teat–raw milk. Evidencing these microbial transfers from grassland ecosystem to raw milk in a causal relationship is therefore critical to better understand the “terroir” effect, i.e. the drivers relating the specificity of a product to its area of production. In addition, it would clearly bridge grassland ecosystem diversity (plants and microorganisms) to the characteristics of dairy PDO products.

Over the past decades, PDO dairy productions experienced a strong economic development leading to technological and zootechnical developments such as the increase of herd size and dairy production, and consequently to an increasing need in forage quantity. To increase forage production in grasslands, a commonly used leverage is grassland fertilization mainly through farmyard manure but also industrial fertilizers. These quantitative and qualitative variations of fertilizers affect soil and phyllosphere microbial communities^25–28^ and more particularly phyla also observed frequently in raw milk or on cow-teat: *Actinobacteria, Proteobacteria* and *Firmicutes*^28–30^. Observed among various environmental conditions, they are involved in organic matter degradation, but also cheese flavor during ripening and potentially to cheese quality^20^. Fertilizers also affect plant community composition^1,31,32^: fertilization can increase plant species richness in nutrient-poor grasslands^33^ but high fertilizer inputs could dramatically reduce plant species diversity in grasslands by favoring fast-growing species at the expense of less competitive ones^34^. Consequently, evidencing a causal relationship based on microbial transfers from grassland ecosystem to raw milk to understand the “terroir” effect also requires the evaluation of its dependency on fertilization practices, i.e. whether fertilization practices affect microbial transfers from grassland ecosystem to raw milk.

The objectives of this study were to evaluate the microbial transfers from grassland compartments (soil and phyllosphere) to cow-teat and raw milk, and identify environmental drivers determining these transfers. To reach these objectives, PDO Comté cheese dairy production was considered as a case study and a network of 44 farms was constituted at the scale of PDO Comté area (2300 km^2^) in the French Jura Mountains. Across this farm network, metabarcoding (Illumina®) was used to characterize prokaryotic (bacteria and archaea) and fungal diversity in the four compartments of the sequence soil–phyllosphere–cow-teat–raw milk. Agricultural practices on grasslands and herd management were characterized in each farm by means of inquiries. Based on these characterizations, a network analysis was used to evaluate the links between the sequence compartments based on correlation coefficients and the composition of microbial communities (genera relative abundance), each far being a replicate. A weighted topological network approach was used to identify a consensus network based on farm replicates since Comté cheese is produced after mixing milks from multiple farms. Variations of the correlation coefficient of significant links identified in the consensus network were used to evaluate the dependency of these links to environmental drivers and agricultural practices at the farm network level. Significant links between compartments can be interpreted as plausible microbial transfers from one compartment to another because of the experimental design and farmers practices.

## Results

### Description of farms: grassland sites characterization (soils, plant community variables), grassland and herd management

Farms involved in the network were distributed all along the elevation gradient ranging from 328 to 1238 m, the average elevation being 806.5 m a.s.l. and 50% of the farms were located at an elevation between 640 to 940 m a.s.l. (Table 1). Soil physico-chemical characteristics were highly variable (Table 1): Soil texture varied between silty/silty loam and silty clay/clayey categories in USDA classification, soil organic carbon content (C_org_) ranged from 27 to 103 g kg^−1^ of dry soil (mean C_org_: 57.6 g kg^−1^ of dry soil); while C:N ratio ranged only from 8.7 to 10.8 (mean C:N ratio: 9) and soil pH ranged from 5.5 to 7.9 (mean pH: 6.7).

**Table 1:** Summary statistics of environmental conditions and grassland and herd management.

Type	Variable	Mean (± SE)	min	max	CV (%)
Climate	Elevation (m a.s.l.)	806.5 (± 35.1)	328.6	1237.9	0.3
Soil	C_org_ (g kg^−1^)	57.6 (± 2.6)	27.0	103.0	0.3
	N_tot_ (g kg^−1^)	5.9 (± 0.3)	2.8	10.7	0.3
	CaCO_3_ (g kg^−1^)	30.6 (± 10.1)	0.5	343.0	2.2
	C:N ratio	9.7 (± 0.1)	8.7	10.8	< 0.01
	pH	6.7 (± 0.1)	5.5	7.9	0.1
	P_2_O_5_ (g kg^−1^)	4.4 10^–2^ (± 2.7 10^–3^)	1.5 10^–3^	0.1	0.4
	NO_3_ (mg kg^−1^)	18.9 (± 2.5)	1.6	95.3	0.9
	NH_4_ (mg kg^−1^)	15.3 (± 0.5)	8.2	25.1	0.2
	Clay (%)	37.8 (± 1.7)	19.4	66.3	0.3
	Silt (%)	52.4 (± 1.4)	32.6	68.2	0.2
	Sand (%)	9.8 (± 1.3)	1.1	35.9	0.9
Vegetation	Plant species richness (SpRichness)	32 (± 2)	12	58	0.3
	Pielou evenness	2.4 10^–1^ (± 7.7 10^–3^)	1.33 10^–1^	3.7 10^–1^	0.2
	Grass (%)	62.0 (± 1.5)	41.0	91.5	0.2
	Forb (%)	19.1 (± 1.3)	1.1	37.2	0.4
	Legume (%)	18.9 (± 1.3)	3.6	45.9	0.5
Grassland management	Cattle (LU d ha^−1^)	392.0 (± 24.8)	123.0	779.1	0.4
	Cattle_spring (LU d ha^−1^)	126.7 (± 9.8)	0	294.6	0.5
	Cattle_summer (LU d ha^−-1^)	144.8 (± 12.1)	0	385.7	0.6
	Cattle_autumn (LU d ha^−1^)	120.4 (± 9.7)	0	249.4	0.5
	P_ind (kg P ha^−1^ year^−1^)	1.7 (± 0.6)	0	18.5	2.3
	K_ind (kg K ha^−1^ year^−1^)	4.6 (± 1.9)	0	75	2.7
	N_ind (kg N ha^−1^ year^−1^)	14.1 (± 2.7)	0	68	1.3
	Liquid_manure (kg N ha^−-1^ year^−1^)	11.1 (± 2.0)	0	50.6	1.2
	Solid_manure (kg N ha^−1^ year^−1^)	0.8 (± 0.3)	0	12.1	2.9
	Total_manure (kg N ha^−1^ year^−1^)	11.9 (± 2.0)	0	50.6	1.1
	Total_N_fertilization (kg N ha^−1^ year^−1^)	25.9 (± 3.6)	0	90.6	0.9
	Manure_prop	0.6 (± 0.1)	0	1	0.7
Herd management	Dairy_cows	64 (± 6)	25	200	0.6
	Milking_preparation	None: 1/dry: 26/humid: 17			
	Cow-teat_care	Yes: 35/no: 9			

C_org_: Soil organic carbon content (g kg^−1^); N_tot_: Total soil nitrogen content (g kg^−1^); CaCO_3_: Soil carbonate content (g kg^−1^); C:N ratio: Soil organic carbon to nitrogen ratio; pH: Soil pH in water; P_2_O_5_: Soil phosphate content (g kg^−1^); NO_3_: Soil nitrate content (mg kg^−1^); NH_4_: Soil ammonium content (mg kg^−1^); Clay: Clay content (g kg^−1^); Silt: Silt content (g kg^−1^); Sand: Sand content (g kg^−1^); Grass: relative cover of grasses (graminoids) in the plant community (%); Forb: relative cover of forbs in the plant community (%); Legume: relative cover of legumes in the plant community (%); Cattle: Annual grazing pressure expressed in livestock units equivalent day per hectare (LU d ha^−1^); Cattle_spring: Spring grazing pressure expressed in livestock units day per hectare (LU d ha^−1^); Cattle_summer: Summer grazing pressure expressed in livestock units day per hectare (LU d ha^−1^); Cattle_autumn; Autumn grazing pressure expressed in livestock units day per hectare (LU d ha^−1^); P_ind: Annual P fertilization by means of industrial fertilizers (kg P ha^−1^ year^−1^); K_ind: Annual K fertilization by means of industrial fertilizers (kg K ha^−1^ year^−1^); N_ind: Annual N fertilization by means of industrial fertilizers (kg N ha^−1^ year^−1^); Liquid manure: Annual N fertilization by means of liquid manure (kg N ha^−1^ year^−1^); Solid manure: Annual N fertilization by means of solid manure (kg N ha^−1^ year^−1^); Total manure: Annual N fertilization by means of manure (kg N ha^−1^ year^−1^); Total N fertilization: Sum of annual nitrogen fertilization by means of industrial fertilizers and manure (kg N ha^−1^ year^−1^); Manure prop: proportion of manure in total annual nitrogen fertilization; dairy cows: Number of dairy cows in the farm; milking preparation: preparation of cow-teat before milking; cow-teat care: protection of cow-teat after milking. Sample size: n = 44.

Plant communities were highly variable. Plant species richness (SpRichness) ranged from 12 to 58 species (mean SpRichness: 32.0 (± 1.7 SE) species). In many plots, plant communities were dominated by few plant species, such as *Lolium perenne*, *Trifolium repens*, *Agrostis capillaris*, *Dactylis glomerata*, *Festuca pratensis*, *Taraxacum officinale*, *Poa trivialis*, *Festuca rubra* and *Ranunculus acris*, since species Pielou evenness was low and ranged from 0.1 to 0.4. Most of the plant communities were dominated by grasses (41.0% to 91.5% relative cover), forbs and legumes being equally represented in plant communities (average: 19.1% and 18.9% relative cover).

Grassland management was characterized first according to grazing pressure at an annual scale (Cattle) and across the 3 grazing seasons (Cattle_spring, Cattle_summer, Cattle_autumn). Cattle ranged from 123 to 779 LU d ha^−1^ with an average of 392 LU d ha^−1^ (a livestock unit LU is equivalent to a dairy cow). Cattle was the sum of Cattle_spring, Cattle_summer and Cattle_autumn which ranged from 0 to 294.6, 0 to 395.7 and 0 to 249.4 LU d ha^−1^, respectively. Among seasons, average grazing pressures were not significantly different. Fertilization practices were considered in terms of nitrogen, phosphorous and potassium inputs from industrial mineral fertilizers (N_ind, P_ind, K_ind), and in terms of available nitrogen inputs from farmyard manure (Liquid_manure, Solid_manure, Total_manure). Nitrogen inputs from industrial fertilizers were significantly higher than those from solid manure (*P* < 0.001, Wilcoxon paired test), but similar to those from liquid manure or total manure (sum of liquid and solid manure). This led to an average total N fertilization (total_N_fertilization) of 25.9 kg N ha^−1^ year^−1^, in which the proportion of nitrogen from farmyard manure ranged from 0 to 1 (mean Manure_prop: 0.6 ± 0.1).

Herd management was characterized by the number of dairy cows (Dairy_cows), milking preparation (Milking_preparation) and cow-teat care (Cow-teat_care, post dipping of teats). Dairy_cows ranged from 25 to 200 with an average dairy herd size of 64 ± 6 cows. Milking_preparation followed three modalities unequally represented: Milking_preparation was represented by a humid way to prepare cow-teats for milking through water or soap solutions in 26 farms, while in all other farms except one, it corresponded to a dry way through hay, wood wool or paper towels (17 farms). After milking, cow-teats were treated in 35 farms out of 44 mainly with bactericide or bacteriostatic (e.g. iodine or mixture of sodium chlorite and lactic acid; no antibiotics) to preclude mastitis (Cow-teat_care).

### Comparison of microbial community composition across compartments

The comparison of microbial community composition across compartments of the sequence soil–phyllosphere–teat–milk was performed at the farm network level based on genera relative abundance data, farms being considered as replicates. Microbial communities of each compartment in the sequence soil–phyllosphere–teat–milk were compared to each other according to their composition and their richness in terms of prokaryotic and fungal genera. Finally, co-occurrence of genera between the compartments of the sequence soil–phyllosphere–teat–milk was evaluated by merging lists of observed genera per compartment among farms. Nevertheless, information on the frequency at which genera co-occurred at the farm level are also provided (Supplementary Data 2).

#### Prokaryotic and fungal communities across farm compartments

**Figure 1:**
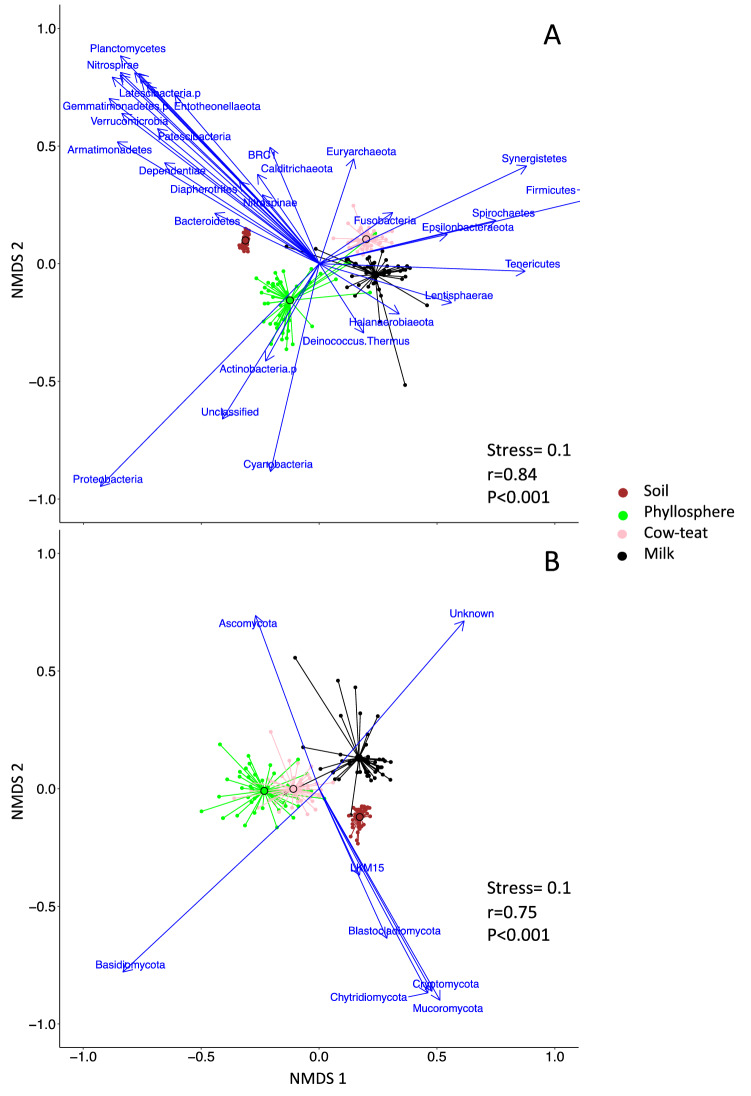
NMDS representation of bacterial-archaeal (**A**) and fungal (**B**) community structure variations across compartments based on the relative abundance of affiliated genera data. For each compartment, each sample from one farm is related to the centroid of the compartment (black lined dot), the total number of samples per compartment was n = 44. Blue arrows represent phylum data fitted by means of *envfit* function (*vegan* package) in NMDS space for which *P* < 0.001. Stress value indicate the quality of NMDS representation; r and *P* values correspond to the results of the ANOSIM analysis.

Microbial communities were compared for their composition (i.e. relative abundance) at the genus level across the four farm compartments of the sequence by means of a Non-Metric Multidimensional Scaling (NMDS) approach. Mean relative abundance of observed genera per compartment (soil, phyllosphere, cow-teat, milk) are provided in Supplementary Data 1. Results are presented in Fig. 1 in which the four farm compartments are compared for their prokaryotic or fungal community composition (Fig. 1A,B, respectively).

Prokaryotic communities were discriminated from one another according to the farm compartment (Fig. 1A). According to centroids position, prokaryotic communities from soil and phyllosphere were discriminated from those of milk and cow-teat along NMDS1 axis. Along NMDS2 axis, soil and phyllosphere prokaryotic communities were discriminated from one another whereas cow-teat and milk communities were only slightly distinguishable. This discrimination between soil, phyllosphere and cow-teat/milk was confirmed by the ANOSIM analysis (a.k.a. Analysis of Similarities; r = 0.84, *P* < 0.001). Prokaryotic communities of cow-teat and milk were discriminated from the others by a higher relative abundance of genera related to dominant or rare phyla: *Firmicutes* (e.g.* Romboutsia*, *Mogibacterium*, *Staphylococcus*, *Turicibacter*, *Acetitomaculum; 0.6% to 6%*); *Spirochaetes* (e.g.* Sphaerochaeta*, *Sediminispirochaeta*, *Treponema;* < *0.1%*); *Fusobacteria* (e.g.* Fusobacterium*, *Cetobacterium*, *Caviibacter*, *Hypnocyclicus;* < *0.1%*). Phyllosphere was characterized by higher relative abundance of *Proteobacteria* (e.g.* Methylobacterium, Massilia, Sphingomonas, Variovorax, Pseudomonas;* > *1.5%), Actinobacteria* (e.g.* Nocardioides*, *Rhodococcus;* > *1%*) or *Cyanobacteria* (e.g. *Scytonema*, *Cyanothece*, *Xenococcus;* < *0.1%*). Soil exhibited higher relative abundance of numerous phyla, especially *Bacteroidetes* (e.g. *Flavobacterium*, *Terrimonas*, *Chitinophaga*, *Ferruginibacter;* > *1.4%*), Thaumarcheota (e.g. *Candidatus Nitrocosmicus*, *Candidatus Nitrososphaera*; > 1.9%), *Acidobacteria* (*Vicinamibacter, Holophaga, Geothrix , Thermoanaerobaculum*; < 0.1%), *Planctomycetes* (*Pirellula*, *Gemmata*, *Fimbriiglobus*, *Tepidisphaera*; > 0.5%) or *Nitrospirae* (*Nitrospira*; > 0.8%). These observations were confirmed by LEfSe and LDA analyses (Supplementary Information S1 and S2). This analysis highlighted that regarding to the other compartments of the sequence, soil was characterized by higher levels of *Acidobacteria*, *Planctomycetes*, *Nitrospirae*, *Thaumarchaeota* or *Delta-proteobacteria*; while phyllosphere was characterized by *Cyanobacteria*, *Actinobacteria* and *Proteobacteria*; and that Clostridia and Bacilli (*Firmicutes*) or *Flavisolibacter* (*Bacteroidetes*) were highly abundant in milk and on cow-teat.

Fungal communities were discriminated according to the farm compartment (Fig. 1B). According to centroids position, fungal communities from phyllosphere and cow-teat were discriminated from soil and milk along NMDS1 axis, while soil was discriminated from milk along NMDS2 axis. Cow-teat and phyllosphere exhibited similar fungal communities; as confirmed by ANOSIM analysis (r = 0.75, *P* < 0.001). Milk compartment exhibited higher relative abundance of *Ascomycota* (e.g.* Scheffersomyces*, *Geotrichum, Pichia*; > 2.5%) whereas phyllosphere and cow-teat compartments were discriminated by *Basidiomycota *(e.g.* Vishniacozyma, Cladosporium, Phaeotremella, Filobasidium;* > *3%*). Soil exhibited higher relative abundance of multiple phyla, especially *Glomeromycota* (recently *Mucoromycota*; e.g.* Glomus, Mortierella*; > 1.5%) and *Chytridimycota* (*e.g. Catenomyces*, *Pseudorhizidium*; < 0.1%). LEfSe and LDA analyses confirmed these results (Supplementary Information S3 and S4). Soil had higher relative abundance of *Chytridiomycota*, *Blastocladiomycota*, *Agaricomycetes* (*Basidiomycota*) or *Pezizomycetes* and *Sordariales* (*Ascomycota*) while *Ustillaginomycetes* and *Tremellomycetes* (*Basidiomycota or Taphrinomycetes* (*Ascomycota*) were highly abundant in the phyllosphere. *Neocallimastigomycota* (*Basidiomycota*), *Leotiomycetes* and *Glomerellales* (*Ascomycota*) were more abundant on cow-teats while *Saccharomycetes* (*Ascomycota*) or *Trichosporonales* (*Basidiomycota*) were mainly observed in milk.

#### Prokaryotic and fungal communities shared taxa across farm compartments

Across the 1917 prokaryotic and 1080 fungal genera observed, the respective number of genera identified in each farm compartment was in the same order of magnitude (Table 2). The average number of observed prokaryotic genera was 424 in soil, 454 in phyllosphere, 407 on cow-teats and 378 in milk. For fungi, the average number of observed genera was 284 in soil, 208 in phyllosphere, 264 on cow-teats and 140 in milk. Differences in terms of genera number were not significant (Spearman rank test) except between cow-teat and soil or cow-teat and milk for prokaryotic communities. This was mainly related to the large range and variability of the number of genera in each compartment (Table 2).

**Table 2:** Summary statistics of genera number by compartment.

	Soil	Phyllosphere	Cow-teat	Milk
**Prokaryotic (bacteria and archaea)**				
[min; max]	[371;481]	[283;599]	[302;486]	[164;641]
mean	424	454	407	378
SE	4	11	8	13
CV (%)	6	15	12	22
Cumulated number of genera	1223	1422	1295	1373
**Fungal**				
[min; max]	[205;356]	[151;262]	[189;369]	[90;528]
mean	284	208	264	140
SE	5	4	5	11
CV (%)	12	14	14	50
Cumulated number of genera	856	751	848	726

**Figure 2:**
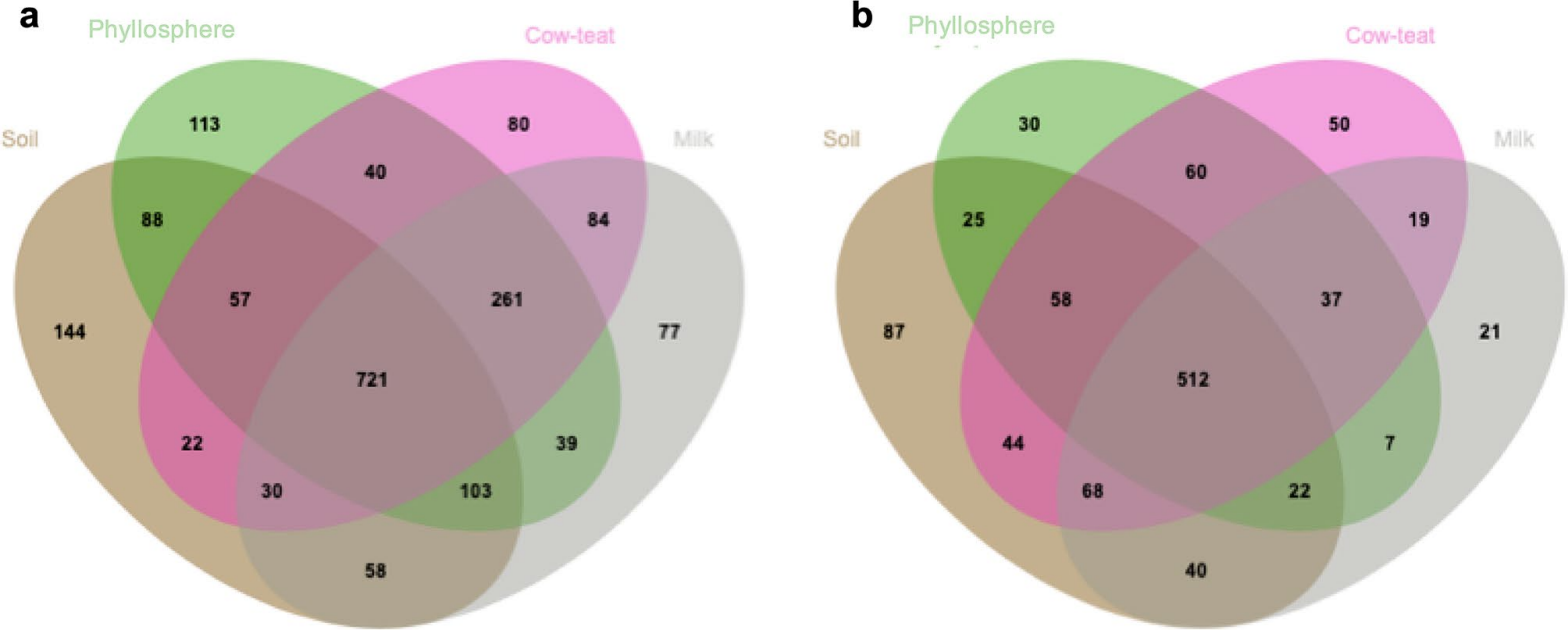
Venn diagrams of bacterial-archaeal (**a**) and fungal communities (**b**) over the 44 farms. Each number represents a number of genera shared by two, three or four compartments when their respective areas overlap; or specific to a compartment in non-overlapping areas.

Venn diagrams (Fig. 2A,B, Supplementary Data 1) represent the number of genera specific or co-occurring in the different compartments for prokaryotes or fungi. No discrepancy was observed for the cumulated number of genera per farm compartment for prokaryotes (soil: 1223, phyllosphere: 1422, cow-teat: 1295, milk: 1373) or fungi (soil: 856, phyllosphere: 751, cow-teat: 848, milk: 726). At the farm network level, the proportion of co-occurring genera tended to be higher when multiple compartments were considered (Fig. 2): between four farm compartments: 721 prokaryotic genera (37.6%) and 512 fungal genera (47.4%); between 2 or 3 compartments: 17.3% and 23.5% of prokaryotic genera, 18.1% and 17.1% for fungal genera; only in 1 compartment: 21.6% of prokaryotic genera and 17.4% of fungal genera. Co-occurring genera belonged to dominant microbial phyla in some compartments such as *Firmicutes* (*Streptococcus*, *Staphylococcus*, *Lactococcus*, L*actobacillus*) or *Ascomycota* (*Geotrichum*); but also to minor phyla or subphyla: e.g.* Planctomycetes* (28 genera, e.g.* Blastopirellula*, *Planctomicrobium*, *Telmatocola*), *Chloroflexi* (23 genera, e.g.* Nitrolancea*, *Sphaerobacter*); or *Glomeromycotina* (7 genera; e.g.* Glomus*, *Paraglomus*, *Archaeospora*), *Blastocladiomycota* (*Catenaria*) with some genera being associated to rind and paste of the cheeses such as Comté (e.g.* Brevibacterium, Corynebacterium, Lactococcus, Lactobacillus, Microbacterium, Micrococcus, Staphylococcus, Streptococcus, Saccharomyces, Sporobolomyces, Pichia, Candida, Geotrichum*). Nevertheless, some genera were only detected in particular compartments: phyllosphere: e.g. Hydrogenobacter (*Aquificae-p*); *soil: *e.g.* Caldithrix, Calorithrix (Calditrichaeota), Nitrospina (Nitrospinae), Mesoaciditoga (Thermotogae-p); cow-teat: Dictyoglomus (Dictyoglomi)*.

### Evaluation of microbial transfers from permanent grassland ecosystems to milk and their dependence to environmental conditions

**Figure 3:**
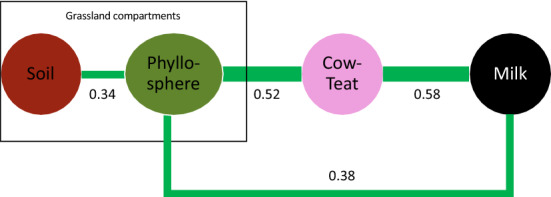
Network analysis of compartment relationships according to their microbial compositions (prokaryotic and fungal). Circles represent compartments and line the significant links derived from the WTO analysis. Line thickness is proportional to link strength as measured by the coefficient of correlation between compartments.

Microbial transfers from permanent grasslands to milk along the sequence soil–phyllosphere–cow-teat–milk were evaluated at the farm network level by means of a network approach based on genera relative abundance, each farm being a replicate. A weighted topological network approach was used to identify a consensus network based on farm replicates (Fig. 3). At the 0.1% probability level, the consensus network highlighted significant links between soil and phyllosphere, phyllosphere and cow-teat, cow-teat and milk, and between phyllosphere and milk; which represented plausible microbial transfers between these compartments. All links presented a correlation coefficient higher than 0.34, the highest being observed between cow-teat and milk and cow-teat and phyllosphere.

To better understand these relationships, variations of correlation coefficients between compartments were confronted to environmental conditions and agricultural practices by means of a multiple linear regression approach. Environmental conditions involved geomorphology, soil characteristics, plant community characteristics, grassland and herd management (Table 3). Multiple linear model explained 48% of variations in microbial transfers between soil and phyllosphere, highlighting that these transfers were significantly modulated by farmyard fertilization (Total_manure 14.8%), plant species richness (SpRichness, 8.8%), grass relative cover (Grass, 12.1%) and soil pH (12.6%). Soil pH and total manure had a polynomial effect (degree 2). Partial derivation allowed estimating the *optimum* of pH to 7.1 and of farmyard fertilization to 29.8 kg N ha^−1^ year^−1^. Microbial transfers increased with increasing pH or total manure below their *optimum* (Supplementary Information S5) whereas it decreased above their *optimum*. Increasing plant species richness and grass relative cover increased microbial transfers from soil to phyllosphere.

**Table 3:** Effects of environmental conditions on microbial transfers.

Link	Explanatory variable	Standardized Regression coefficient	Variance (%)	R^2^	R^2^_adj_	*P* value
Soil–phyllosphere	Total_manure**	1.181	9.7	0.48	0.40	0.003
	pH*	6.085	6.3			
	SpRichness**	4.119 × 10^–1^	8.8			
	Total_manure^2^*	− 9.087 × 10^–1^	5.1			
	pH^2^*	− 5.810	6.3			
	Grass^2^**	3.533 × 10^–1^	12.1			
	Residuals		51.7			
Phyllosphere–cow-teat	Cattle_spring**	5.753 × 10^–1^	5.9	0.21	0.16	0.021
	Elevation^2^: cattle_spring*	− 3.752 × 10^–1^	8.8			
	Total_manure*	− 3.113 × 10^–1^	6.8			
	Residuals		78.5			
Cow-teat–milk	Cattle_spring*	1.053	4.7	0.34	0.27	0.002
	Cattle_spring^2^*	− 1.104	13.5			
	Cow-teat care*	− 2.795 × 10^–1^	8.6			
	Cattle_spring^2^: total_manure^2^*	− 3.151 × 10^–1^	7.0			
	Residuals		66.2			
Phyllosphere–milk	Cattle_summer*	9.310 × 10^–1^	0.1	0.11	0.07	0.094
	(Cattle_summer)^2^*	− 1.014	10.8			
	Residuals		89.1			

R^2^, R^2^_adj_ and *P* value refer to the whole model. The significance of the marginal effect of each variable is indicated by the following codes: *P < 0.05; **P < 0.01, ***P < 0.001.

Multiple linear model for microbial transfers between phyllosphere and cow-teat explained 21% of their variations. The first variable explaining these variations was Total_manure (6.8%) followed by Cattle_spring (5.9%) and interactions between elevation and Cattle_spring (8.8%). Total_manure and Cattle_spring had opposite effects on microbial transfers.

Multiple linear model explained 34% of the variations in the microbial transfers from cow-teat to milk. The environmental factors explaining these variations were Cow-teat_care (8.6%), Cattle_spring (18.2%) and Total_manure in interaction with Cattle_Spring (7.0%). Cattle_spring had a polynomial effect with an *optimum* estimated up to 137 LU d ha^-1^ by partial derivation (Supplementary Information S6). Below this *optimum*, microbial transfers increased with Cattle_Spring and decreased with increasing Cattle_Spring above the *optimum*.

Transfers between phyllosphere and milk were identified significant but were poorly explained by the environmental variables since only 10% of their variations were explained by Cattle_summer, the summer grazing pressure.

## Discussion

In this study, the farm network covered the complete altitudinal gradient and the different soil types observed in the PDO Comté cheese area. Selected grasslands were diversified in terms of soil physico-chemical characteristics, fertilization management and grazing pressure. The farm sample was representative of the practices in the PDO Comté cheese area for herd management even if it was not balanced regarding teat preparation before milking or cow-teat care after milking.

To characterize prokaryotic communites through metabarcoding, the V3-V4 region of 16s rRNA gene was used. Even if it did not allow OTU identification at the species level in regards of other less conservative regions/other target genes, it provided a good consensus between taxonomic resolution (genus) and specificity for prokaryotes (*Bacteria* and *Archaea*) while excluding eukaryotes^35^.

At the genus level, the taxonomic richness observed in soil samples was in the range of those observed in the literature for grassland ecosystems either for prokaryotes or fungi^3,36,37^. Publications relative to phyllosphere microbial communities mainly focus on particular species and are quite scarce for grasslands. Nevertheless, they report high levels of microbial diversity, since the number of OTUs observed at the genus level ranged from 100 to 1000 OTUs either for prokaryotes or fungi^25,37,38^, which is in the order of magnitude observed here for taxonomic richness of phyllosphere. Similarly, the taxonomic richness on cow-teat or in milk were high, in agreement with recent studies highlighting the diversity of teat and milk microbiomes^21–23^. However, the range observed was larger than in the literature. This may be related to the diversity of ways and materials used for milking preparation, two factors inducing changes in teat and milk microbial diversity^21–23^. This high diversity may also be related to the large number of farms studied here (n = 44) since lower microbial diversity was observed for samples from a single farm^21,22,39^, independently of season, breed or feeding regime. These high prokaryotic and fungal richness at the genus level were not associated to particular phyla since sequences were affiliated to at least 25 prokaryotic phyla and 9 fungal phyla or subphyla in each compartment of the sequence soil–phyllosphere–cow-teat–milk, as observed in the literature^3,22,23,25,40–42^.

The different compartments were compared for their microbial communities’ compositions at the genus level, highlighting that prokaryotic and fungal communities significantly differed between soil, phyllosphere, cow-teat and milk based on the relative abundance of genera and their occurrence in each compartment. For prokaryotes, soil was mainly discriminated from other compartments by genera belonging to phyla classically observed in soil microbial ecology studies as major or abundant taxa in soils (*Acidobacteria*, *Bacteroidetes* and *Planctomycetes*, *Nitrospirae* and *Chrenarchaeota, Delta-Proteobacteria*)^29,40,43^ and also by *Nitrospina* (*Nitrospinae*) known to be a nitrite oxidizing bacteria in soil. Phyllosphere presented high levels of genera related to *Proteobacteria* (*Alpha-Proteobacteria and Gamma-Proteobacteria*) which are tolerant to thermic and water stress but also include N-fixing taxa, in agreement with the literature^25,38^, and *Cyanobacteria* which can be exposed to light when located on grass leaves or *Actinobacteria* which are considered as ubiquitous organisms with a high diversity of ecological niches, notably plant tissues^44^. Cow-teat and milk were notably characterized by high levels of genera related to *Firmicutes*, which is classically observed^22,23,39^, especially in spring and autumn^45^. For fungi, the discrimination was mainly based on three phyla, which were the more abundant in the soil: *Glomeromycota* (recently *Mucoromycota*), *Basidiomycota* and *Ascomycota* producing fruiting bodies. Conversely, non-fruiting *Basidiomycota* were more abundant in the phyllosphere and on cow-teats^44^ while non-fruiting *Ascomycota* were more abundant in milk. This could be related to the symbiotic relationships between plant roots and *Glomeromycota* taxa, or to the reproduction cycle of the subphyla of *Basidiomycota* and *Ascomycota* producing fruiting bodies. *Ascomycota* classically observed in milks are often related to yeast taxa^42,46^, in agreement with the subphyla observed here; and *Basidiomycota* have already been identified on plant leaves^37,42^. Therefore, microbial communities were consistently characterized, supporting testing for microbial transfers along the compartments of the sequence at the genus level.

First, a co-occurrence analysis highlighted that *ca.* 37% to 47% of the identified genera were shared by the four compartments from soil to milk. These genera were affiliated to 17 prokaryotic phyla and 7 fungal phyla. This high level of co-occurrence was mainly associated to genera related to dominant phyla such as *Streptococcus*, *Staphylococcus*, *Lactococcus*, *lactobacillus* or *Geotrichum;* but also to some minor phyla: *Blastopirellula* , *Planctomicrobium*, *Telmatocola*, *Nitrolancea*, *Sphaerobacter*, *Glomus*, *Paraglomus*, *Catenaria*. Other studies already highlighted this high level of co-occurrence between cow-teat and milk^22,39^ but never between soil and the downstream compartments of the sequence to raw milk. In addition, some of these genera were already identified as playing a role in cheese making^22^. Altogether, this emphasizes that raw milk microbiota may find their origin, at least partly, in the grassland ecosystem; a hypothesis supported by previous studies^22,47^. To better evaluate this hypothesis, a network analysis has been conducted at the farm network level in which compartments in the sequence soil–phyllosphere–cow-teat–milk were related by means of correlation coefficients based on their respective microbial communities’ compositions (genus level). It highlighted significant links all along the sequence soil–phyllosphere–cow-teat–milk, and more surprisingly between phyllosphere and milk. These links can be interpreted as plausible microbial transfers since sampling design supports downstream relationships along the sequence: cows spent all their time in pastures in spring; cow-teats and milk samples were collected once cows grazed the selected grassland plot; and farmers daily cleaned milking equipment. Downstream transfers from soil to phyllosphere, to cow-teat and then to milk are easily related to successive contacts between the different compartments through animals or milking, or by potential transfers through aerosols originating from cow skin or hair^19^. As far as we know, the literature on these transfers is very restricted, limiting comparisons. Nevertheless, studies supporting the transfers between cow-teat and milk support these results^19,22,47^. Identifying a link between phyllosphere and milk was more surprising but can be explained by the large core microbiota shared by phyllosphere, cow-teat and milk (982 prokaryotic genera, 549 fungal genera) regarding to their “specific” microbiota (77 to 113 prokaryotic genera, 21 to 50 fungal genera).

Since network analysis was based on replicated estimations of links strength in the farm network, it allowed evaluating of their dependency to environmental factors, grassland or herd management. Transfers between soil and phyllosphere were related to grassland vegetation: plant species richness and grass relative cover, but also to soil pH and farmyard fertilization level. Altogether, these effects could be explained by the importance of these factors in defining microbial ecological niches in soil or phyllosphere. The diversification of plant communities may increase the number of foliar niches^37,38,48^ and therefore increase the probability for a soil microbe to find suitable conditions for its implantation or maintenance on leaves. Biogeographic studies identified soil pH as a crucial component of the ecological niches in soil for microbes^36,49^ with bacterial richness increasing with soil pH up to an *optimum* for soil bacteria (7.5–8). This *optimum* was close to the one estimated in this study. Therefore, soil pH could modulate the pool of soil microbes potentially transferred from soil to phyllosphere. Similarly, fertilization of grasslands with manure has been identified to modulate the composition of soil and phyllosphere microbial communities^25,27,28^, together with plant community composition^1,33^. This relationship also presented an *optimum* of *ca.* 30 kg N ha^−1^ year^−1^. Therefore, the pool of microbes potentially transferred from soil to phyllosphere can similarly be modulated by the level of grassland fertilization. Manure fertilization and grazing pressure in spring, but also the interaction between grazing pressure in spring and elevation, were identified as influencing microbial transfers from phyllosphere to cow-teat. Grazing pressure in spring has been measured as the cumulative time spent by the herd on the grassland plot. Primarily, its positive effect could be understood as an increasing contact between the phyllosphere and cow-teats, leading to an increasing fingerprint of phyllosphere microbiota on cow-teat microbiota. This hypothesis is supported by the plasticity of cow-teat microbiota observed when cow environment is modified, notably from indoor to outdoor^21,22^. This contact hypothesis is also suitable to explain the negative effect of the interaction between elevation and grazing pressure in spring since, in the Jura Mountains context, cows go outdoor later at higher elevations, therefore spending less time on grasslands during spring. But it could also be related to the modulation of the ecological niches available for microbes since grazing pressure is known to induce changes in plant community composition. This niche modulation hypothesis is also suitable to explain the effect of manure fertilization which often leads to increased development of nitrophilous plant species becoming dominant in the plant community^1^. This would reduce the number or evenness of ecological niches and therefore the diversity of microbes to be transferred from phyllosphere to cow-teat. The intensity of microbial transfers from cow-teat to milk were negatively affected by cow-teat care practices after milking, i.e. the use of bactericide or bacteriostatic. These products are known and intended to reduce the development of microbes on cow-teats and to modify their microbial community^21,23,50,51^. The more important may be their degree of remanence which may prevent the fast recolonization of cow-teats by environmental microbial communities when animals go back to the grassland and therefore reduce microbial transfer from cow-teat to milk. Microbial transfers from cow-teat to milk were negatively affected by farmyard fertilization level in interaction with grazing pressure in spring. Increasing the fertilization level may reduce the number of microbial ecological niches at the plot level and therefore reduce the fingerprint of grassland on cow-teat microbiome regarding other environmental sources (hay, bedding, farm buildings) and may be associated to reduced time spent on the pasture by cows. This would be supported by the high proportion of genera shared by phyllosphere, cow-teat and milk. Grazing pressure in spring had a positive effect up to an *optimum* of 137 LU d ha^-1^. This seemed to be in contradiction with the positive effect of grazing pressure on the microbial transfers between phyllosphere and cow-teat but may underline the importance of the time spent by cows on the pasture. Indeed, for a given amount of forage resources, larger herds tend to spend fewer days on the pasture which may reduce contacts between cow-teat and phyllosphere and therefore transfers to milk.

## Conclusion

With a microbial point of view, this study focused on the sequence from permanent grassland to raw milk and identified high levels of prokaryotic and fungal richness in the four considered compartments: soil, phyllosphere, cow-teat and raw milk. It established plausible causal relationships between the compartments of this sequence based on possible microbial transfers between them. The intensity of the transfers, as derived from the similarity between microbial communities, was sensitive to environmental conditions but also to grassland management by means of farmyard fertilization and grazing pressure, and to herd management through cow-teat care. Altogether, this highlights that managing grassland fertilization, grazing pressure and cow-teat care may be relevant leverages to ensure the transfer of microbial communities from grassland plot to raw milk and consequently the presence of the indigenous microbial communities involved in cheese specificity in PDO Comté cheese. Even if these results could be broadened to other PDO cheeses, they support that the “terroir” of dairy PDO products is related to grassland ecosystem diversity (plants, microorganisms). Making "terroir" even more local, this study suggests that this causal relationship has to be considered for sustainable dairy production.

## Material and methods

### Farm network and sampling design

**Figure 4:**
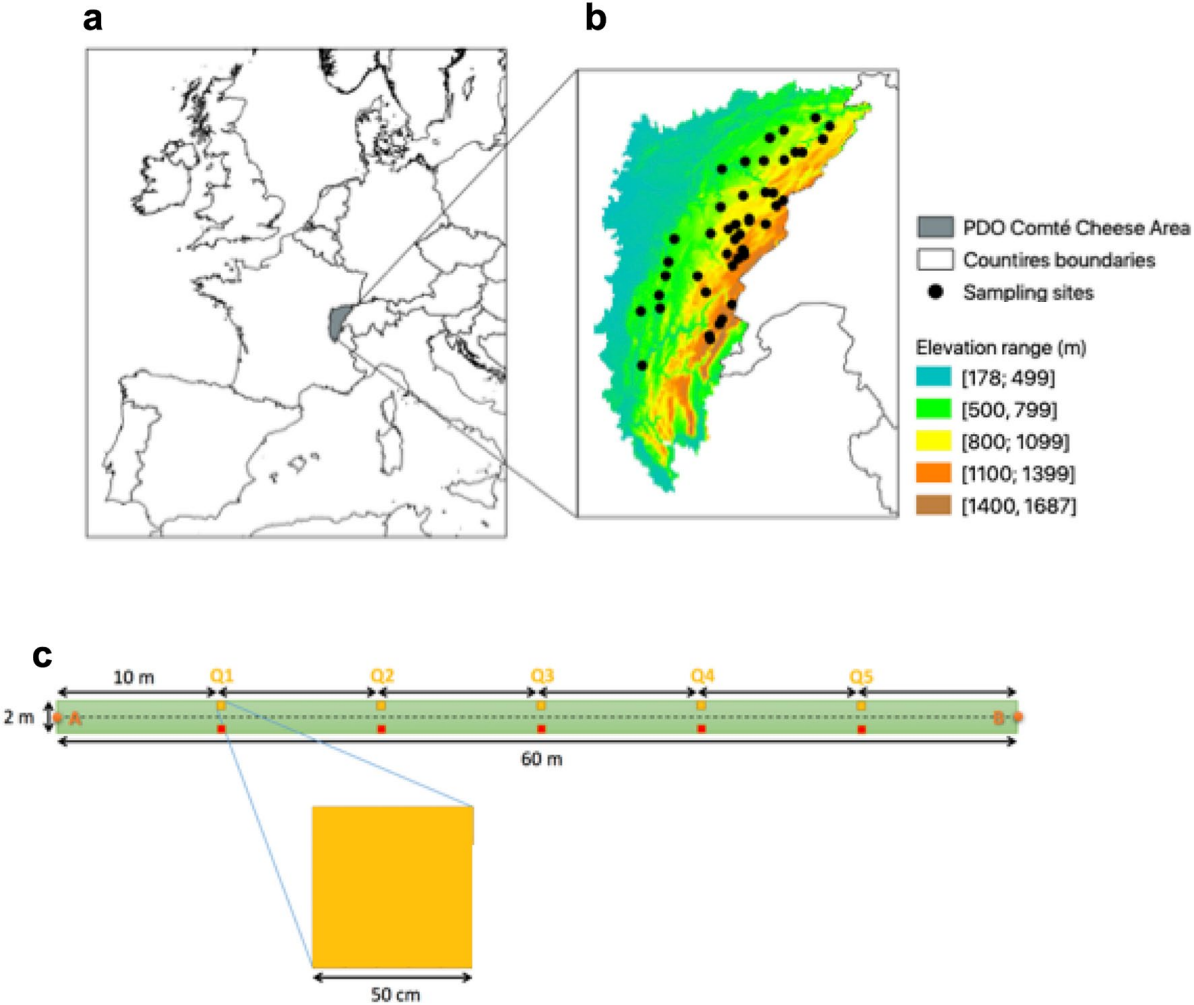
Farm network distribution across the PDO Comté cheese area in the Jura mountains. (**a**) Location of the PDO Comté cheese area. (**b**) Distribution of each farm (black dots, n = 44) in the Jura Mountains. Color gradient provide the elevation information. (**c**) Organization of the sampling area in each grassland showing locations of soil and microbial sampling (red) and quadrats for detailed vegetation records (yellow). Maps in (**a**) and (**b**) were generated under QGis^53^, version 3.12. Background is based on: administrative limits of European countries (https://ec.europa.eu/eurostat/fr/web/gisco/geodata/reference-data/administrative-units-statistical-units/countries) in (**a**); RGE Alti 75 m database (https://geoservices.ign.fr/rgealti) in (**b**). Both databases are open access.

A farm network was constituted based on volunteer farms across the PDO Comté cheese area, the most important PDO cheese in France by its tonnage. This network was constituted of 44 farms distributed all along the French Jura Mountains across an elevation gradient ranging from 328 to 1238 m a.s.l. (Fig. 4A,B) with warm temperate climates at the lowest elevation to boreal climates at the highest elevations^51,52^. The maps presented in Fig. 4A,B were generated by the authors under QGIS 3.12 (https://www.qgis.org)^53^. Background in each map is based respectively on administrative limits of countries (https://ec.europa.eu/eurostat/fr/web/gisco/geodata/reference-data/administrative-units-statistical-units/countries; Fig. 4A) or RGE Alti 75 m database (https://geoservices.ign.fr/rgealti; Fig. 4B), both databases being open access. The area of the French Jura Mountains is characterized by high soil variability with a predominance of cambisols and a relatively high plant diversity in permanent grasslands.

In each farm, four compartments were considered: grassland soil, grassland phyllosphere, cow-teat surface and milk. For this, each farm gave an individual permission for sample collection. First, a parcel of permanent grassland grazed by dairy cows was selected. Within this parcel, a sampling plot of 60 m × 2 m was delimited, located on the flattest area of the parcel far from its margin and georeferenced. Within this sampling plot, two parallel series of five sub-plots (area: 0.25 m^2^) were distributed every 10 m to account for grassland variability (Fig. 4C). Within each subplot of the first series, soil and phyllosphere were sampled using the following procedures. The second series was used for detailed vegetation records. Second, cow-teat surface and milk samples were collected in the milking parlour on cows grazing the selected grassland parcel. For this, at least 10 dairy cows were randomly selected within the herd. Third, the plant community was described over the whole sampling plot (120 m^2^) to account for a maximum of plant species, which is common in studies of permanent grasslands^34,54–56^. All vascular plant species observed in the sampling plot were listed and their cover was estimated according to the Braun-Blanquet dominance scale (r, +, 1, 2, 3, 4, 5). These codes were then converted into absolute percentage cover^55^ and adjusted to relative percentage cover by summing to 100% for each plot. Based on these measures, taxonomic diversity was measured through species richness (SpRichness), Pielou evenness^57^.

The sampling period for soil and phyllosphere ranged from 3 May 2017 to 27 June 2017 and from 27 April 2017 to 29 June 2017 for milk and cow-teat surface. At the farm scale, the time-lag between sample collection in the grassland plot and in the milking parlour ranged from 0 to 8 days. For plant community characterization, the sampling period ranged from May 2017 to July 2017.

### Sampling procedures

At the plot scale, phyllosphere samples were collected before soil samples since soil sampling was destructive. Phyllosphere samples were collected by means of Sterisox Tryptone Kit (Sanifarm, Bolzano, Italie) placed on sterile supports. The two sterisocks were then passed successively on each subplot by taking care of covering the complete area of the subplot. Sterisocks were then placed in a sterile ziplock. In same subplots, two soil cores were sampled using an auger (diameter 5 cm) from 0 to 20 cm depth. Then, all soil cores were mixed together to constitute a composite soil sample which was sieved at 4 mm mesh. All samples were stored at 4 °C until processing in the lab. In the milking parlour, cow-teat samples were taken before teat preparation and the milking on 10 to 20 healthy dairy cows according to herd size by means of sterile wipes representing 10% to 50% of the herd. To do so, for each cow, two teats diagonally distant were wiped and sampling wipe was placed in a sterile ziplock. No live animals were directly handled by researchers during the study and cow-teat samples were collected with each farmer using sterile wipes, wiping cow teats being part of the milking procedure performed by the farmer twice a day on each cow. Milk was sampled directly in the farm milk tank using a sterile ladle at the end of herd milking. Unhealthy or presumed unhealthy cows, *e.g.* having mastisis were being milked apart from the herd and their milk was discarded. All samples were then stored at 4 °C until processing in the lab.

### Environmental conditions and agricultural practices characterization

#### Soil

A subsample of the composite soil sample (500 g) was air-dried at room temperature. Then, soil samples were characterized for their: texture (NF ISO 11277 according to the following granulometry classes in %: Clay: 0–2 µm; Silt: 2–63 µm; Sand: 63–2000 µm); pH in water (NF ISO 10390); organic carbon and total nitrogen contents (g kg^−1^, NF ISO 10694 and NF ISO 13878; respectively); CaCO_3_ content (g kg^−1^, NF ISO 10693); plant assimilable P_2_0_5_ content (g kg^−1^, NF ISO 11263, Olsen method), NH_4_^+^ and NO_3_^-^ contents (mg kg^−1^, INRAe methods). C:N ratio was derived from carbon and nitrogen contents. All analyses were performed by the Laboratoire d’analyse des sols d’Arras (INRAe, https://www6.hautsdefrance.inrae.fr/las) which is Cofrac certificated for these analyses (NF EN ISO/IEC 17025:2107).

#### Agricultural practices

For each of the 44 selected grasslands, farmers provided detailed information about grassland management (grazing and fertilization) and herd management. Description of calculation methods is provided in Table 4. For grassland management, different variables were considered. Grazing pressure was calculated for 2017 for each grazing season (spring, summer, autumn) and annually. Fertilization practices were considered according to the type (industrial fertilizer versus farmyard manure) and the quantity yearly spread on the pastureland. For industrial fertilizers, N, P and K elements were considered (N_ind, P_ind, K_ind in kg ha^−1^ year^−1^). For farmyard manure, the plant available N equivalent amount spread on the parcel was determined (Liquid_manure; Solid_manure; both in kg N ha^−1^ year^−1^) based on manure quantity and mean nitrogen content of the manure type (Table 4) over a 3 years period and averaged. Then, Total_manure (kg N ha^−1^ year^−1^) was calculated as the sum of Liquid_manure and Solid_manure. Total_N_fertilization (kg N ha^−1^ year^−1^) was calculated as the sum of Total_manure and N_ind. Finally, the proportion of nitrogen associated to farmyard manure used (Manure_prop) was calculated by dividing Total_manure by Total_N_fertilization. If Total_N_fertilization was equal to 0, Manure_prop was set to 0.

**Table 4:** Method description for calculating agricultural practices.

Variable	Unit	Description	Equation	Comment
Cattle_spring	LU d ha^−1^	Grazing pressure in spring	$\frac{{LU}_{spring}\times {t}_{spring}}{A}$	LU: number of livestock units (dairy cows) grazing the plot t: time spent by livestock units on the plot in days A: plot Area in ha
Cattle_summer	LU d ha^−1^	Grazing pressure in summer	$\frac{{LU}_{summer}\times {t}_{summer}}{A}$	
Cattle_autumn	LU d ha^−1^	Grazing pressure in autumn	$\frac{{LU}_{autumn}\times {t}_{autumn}}{A}$	
Cattle	LU d ha^−1^	Annual grazing pressure	$Cattl{e}_{spring}+Cattl{e}_{summer}+Cattle\_autumn$	
P_ind	kg P ha^−1^ year^−1^	Amount of phosphorous input from industrial sources per year		
K_ind	kg K ha^−1^ year^−1^	Amount of potassium input from industrial sources per year		
N_ind	kg N ha^-1^ yr^-1^	Amount of nitrogen input from industrial sources per year		
Liquid_manure	kg N ha^−1^ year^−1^	Amount of nitrogen input from farmyard liquid manure per year	$\frac{{V}_{liquid manure}\times Nitrogen content}{A}$	Nitrogen content: Liquid manure: 5 kg N m^−3^ Diluted liquid manure: 3 kg N m^−3^
Solid_manure	kg N ha^−1^ year^−1^	Amount of nitrogen input from farmyard solid manure per year	$\frac{{V}_{solid manure}\times Nitrogen content}{A}$	Nitrogen content: 5 kg N t^−1^
Total_manure	kg N ha^−1^ year^−1^	Amount of nitrogen input from farmyard manure per year	Solid_manure + Liquid_manure	
Total_N_fertilization	kg N ha^−1^ year^−1^	Amount of nitrogen fertilization per year	Total_manure + N_ind	
Manure_prop	no unit	Proportion of nitrogen input originating from farmyard manure	$\frac{\mathrm{Total}\_\mathrm{manure}}{\mathrm{Total}\_\mathrm{N}\_\mathrm{fertilization}}$	0 if Total_N_fertilization equals 0

Herd management was considered by means of dairy cow number, milking preparation and cow-teat care. Milking preparation corresponded to the way cow-teats were cleaned before milking and was characterized according to three categories: None: no cleaning of cow-teat; Dry: Dry cleaning of cow-teat (e.g. hay, wood wool, paper towel); Humid (*e.g.* soap solution, water). Cow-teat care corresponded to the use of a hygiene treatment applied after milking mainly with products were based on iodine or a mixture of sodium chlorite and lactic acid having bactericide or bacteriostatic effects. It has to be noted that no antibiotics were used.

### Microbial community characterization

For each of the four compartments, bacterial-archaeal and fungal communities were characterized by means of culture independent molecular methods.

#### DNA extraction and purification

For soil samples, DNA was extracted from according to the GnS-GII procedure described in Terrat et al.^35^. Briefly, 1 g of lyophilized soil was placed in a 15 ml tube containing 2.5 g of 1.4 mm diameter ceramic beads, 2 g of 106 μm diameter silica beads and four glass beads of 4 mm diameter and an extraction buffer (100 mM Tris pH 8.0, 100 mM EDTA pH 8.0, 100 mM NaCl and 2% (w/v) SDS) in proportion 3:1 (v/w). Tubes were shaken in a FastPrep®-24 (MP-Biomedicals, NY, USA) during 3 cycles of 30 s at 4 m.s-1. Then, tubes were centrifuged at 7000×*g* for 5 min (20 °C) and supernatant was collected. Proteins were precipitated in ice with 1:10 volume of 3 M sodium acetate prior to centrifugation (14,000×*g*, 5 min, 4 °C). Finally, nucleic acids were precipitated by adding 1 volume of ice-cold isopropanol and concentrated into DNA pellets by centrifugation (13000 rpm, 30 min, 4 °C). DNA Pellets were washed with 70% ethanol which was removed by centrifugation (13000 rpm, 5 min, 4 °C) and drying. Finally, the pellet of crude DNA was resuspended in 200 µl water. Finally, crude DNA was purified using Nucleospin soil kit (Macherey–Nagel, France).

For phyllosphere samples and cow-teat surface samples, wipes and Sterisox were washed with an extraction buffer (0.1% of Tween 20® (v/v, Sigma-Aldrich, Steinheim, Germany), 0.5% Lait G powder (m/v), qsp 100 ml with physiological water) in a stomacher for 6 min. The DNA of these suspensions and the DNA from milk samples was extracted with DNeasy PowerFood Microbial kit (Qiagen, Courtaboeuf, France) according to manufacturer’s instructions. Purified DNA was then used for microbial community characterization (procaryotes and fungi) by means of Illumina High-Throughput sequencing.

#### Metabarcoding of bacterial-archeal and fungal communities

First, the DNA concentration was determined using a QuantiFluor staining kit (Promega, USA).

Bacterial-archaeal and fungal communities were characterized using high-throughput sequencing (Illumina MiSeq technology) with the following procedure. For bacterial-archaeal community, a 440-base 16S rRNA fragment was amplified using the F479/R888 primer pair targeting V3–V4 region (5′-CAG CMG CYG CNG TAA NAC-3′/5′-CCG YCA ATT CMT TTR AGT-3′)^7^. For this, 5 ng of DNA were amplified in a 25 µL PCR reaction volume with the following conditions: 2 min at 94 °C; 35 cycles: 30 s at 94 °C, 30 s at 52 °C and 1 min at 72 °C for 1 min; and 7 min at 72 °C for final elongation. For fungal community, a 350-base 18S rRNA fragment was amplified using the primer pair: FF390/FR1 (5′-CGA TAA CGA ACG AGA CCT-3′/5′-ANC CAT TCA ATC GGT ANT-3′)^58^. For this, 5 ng of DNA were amplified in a 25µL PCR reaction volume with the following conditions: 3 min at 94 °C; 35 cycles: 30 s at 94 °C, 1 min at 52 °C, and 1 min at 72 °C; and 5 min at 72 °C for final elongation. All PCR products were purified using AMPure® XP kit (Beckman Coulter, Italy, Milano) and quantified using a QuantiFluor staining kit (Promega, USA). A second PCR was performed on purified amplicons to add multiplex identifiers at the 5- end of the primers so as to allow the specific identification of each sample. For bacterial–archaeal communities, 7.5 ng of 16S rRNA amplicons were used with similar PCR conditions to those described above but only 7 cycles. For fungal communities, 5 ng of 18S rRNA amplicons and optimized PCR conditions were used with 7 cycles and a denaturation step at 94 °C lasting 1 min. PCR products were purified using MinElute purification kit (Qiagen, Courtaboeuf, France) and quantified using a QuantiFluor staining kit (Promega, USA). Finally, samples were pooled in equal amount and cleaned with SPRI (Solid Phase Reverse Immobilization Method) using the AMPure® XP kit (Beckman Coulter, Italy, Milano). The pool was sequenced with a MiSeq Illumina instrument (Illumina Inc., San Diego, CA) operating with V3 chemistry and producing 250 bp paired-reads.

#### Bio-informatic analyses

Bioinformatic analyses were performed with BIOCOM-PIPE^59^. First, the 16S and 18S raw reads (15 881 872 and 15 723 864 raw reads; respectively) were sorted according to each sample using multiplex identifiers. Low quality reads were then deleted based on their length (less than 350-bp for 16S reads and less than 300-bp for 18S reads), their number of ambiguities and their primer(s) sequence(s). Microbial data are accessible on EBI (PRJEB47150). Then, a PERL program was then applied for rigorous dereplication (i.e. clustering of strictly identical sequences). Dereplicated reads were aligned using Infernal alignment^60^ and the number of sequences per sample was normalized (i.e. 10,000 high-quality reads for each sample) by random selection to allow efficient comparison of the data sets and avoid biased community comparisons. Nevertheless, two samples had only 3746 and 9379 high-quality reads and therefore, all high-quality reads were kept for further analyses. For each sample, high-quality reads were then affiliated at the genus level using SILVA database (R132). Affiliation results for 16S rRNA sequences and 18S rRNA sequences were aggregated into 2 contingency tables with samples in rows and genera in columns, each cell containing the number sequences observed for a given genera in the corresponding sample.

### Statistical analyses

#### Comparison of community composition across compartments

Community composition was compared among the four compartments (soil, phyllosphere, cow-teat and milk) for genus occurrence across compartments and for the similarity of community composition between compartments. For the occurrence approach, genus data were converted into presence-absence data and Venn diagrams were constructed using JVenn software^61^. The comparison of community similarity between compartments involved relative abundance data at the genus level and were performed by means of a Non-Metric Multidimensional Scaling approach based on Bray–Curtis distance by means of *metaMDS* function (vegan package)^62^ which standardize the NMDS results. In the NMDS approach, the first two axes were considered. Phylum data were overlaid on the NMDS space by means of the *envit* function (*vegan* package, 1000 permutations) for reading convenience. Differences in community composition between compartments were tested by means of ANOSIM (*anosim* function, *vegan* package) at the 5% probability level. In addition to the NMDS approach, a linear discriminant analysis (LDA) effect size (LEfSe) method^63^ based on the relative abundance of microbial genera was used to evaluate the differences in taxonomic abundance between the four compartments. The cladograms and the LDA list (*P* < 0.05) from the phylum level^64^ to the genus level are presented in Supplementary Materials.

#### Evaluation of microbial transfers across compartments

In microbial ecology, relationships between taxa can be derived from network analyses based either on the co-occurrence or the relative abundance of taxa among sites. In this study, we applied a network analysis to assess if plausible microbial transfers could appear between pairs of farm compartments (soil, phyllosphere, cow-teat and milk). In this approach, farm compartments were considered as nodes of the network and paired variations in the relative abundance of microbial genera (bacteria, archaea and fungi) between two compartments as measures of a possible microbial transfer. Therefore, paired transfers between farm compartments were estimated by means of a Pearson correlation coefficient between genera relative abundance in each compartment and false discovery rate correction^65^ was applied to avoid false positive detection. For the estimation of Pearson correlation between two compartments, all genera absent in both considered compartments were excluded and only significant correlations were conserved (*P* < 0.001). In this analysis, each farm was a replicate and used to estimate of correlation coefficients of the links between pairs of compartments that were merged into a Consensus Network at the farm network level with the *WTO.consensus* function of the *WTO* package^66^. Briefly, this method allows the combination of multiple networks into a consensus one by removing nodes that do not exist in all networks and by estimating a consensus value for each conserved link and the associated *P*-value. For consensus network, the selected threshold for *P*-values was 0.001. Figure 3 presenting the consensus network was created by the authors using Microsoft Powerpoint® 2016.

#### Evaluating the impact of environmental factors on links strength between farm compartments

First, strongly redundant explanatory variables were removed based on their correlation coefficient (r < 0.6). The retained variables were: Elevation, Cattle_spring, Cattle_summer, Total_manure, Corg, pH, Clay, Grass, SpRichness, Dairy_cows, Milking_preparation and Cow-teat_care.

To evaluate which environmental variables significantly impact the strength of links between farm compartments, correlation coefficients between farm compartments determined at the farm level were confronted to environmental explanatory variables by means of a multiple linear regression approach. This approach was chosen instead of Structural Equation Modeling (SEM) approach because sample size was not large enough for SEM. In multiple linear regression approach, quantitative predictors were considered as both their linear and quadratic terms to account for potential optimal or threshold effects, and qualitative variables were converted into dummy variables. First, the set of explanatory variables correlated to the explained variables was refined using a random forest approach based on z-score (*Boruta* function in *Boruta* package)^67^. Then, a stepwise selection procedure based on Akaike Information Criterion was used to identify the most parsimonious model including both marginal effects and interactions. Standardized coefficients were determined by means of lm.beta function (lm.beta package) based on mean and standard error of each variable.

## Supplementary Information

Supplementary Information 1.

Supplementary Information 2.

Supplementary Information 3.
