# Supplementary material for: Microbial transfers from permanent grassland ecosystems to milk in dairy farms in the Comté cheese area
Source: Sci Rep. 2021 Sep 13;11:18144. doi: 10.1038/s41598-021-97373-6 (PMC8438085; doi:10.1038/s41598-021-97373-6)

**Title**

**Microbial transfers from permanent grassland ecosystems to milk in dairy farms in the Comté cheese area**

**Authors:**

Chemidlin Prévost-Bouré N.^1,*^, Karimi B.^1^, Sadet-Bourgeteau S.^1^, Djemiel C.^1^, Brie M.^2^, Dumont J.^2^, Campedelli M.^2^, Nowak V.^1^, Guyot P.^3^, Letourneur C.^3^, Manneville V.^4^, Gillet F.^5^, Bouton Y.^3^

**Supplementary Information**

The LefSe algorithm^66^ was used as a complementary approach to NMDS in order to confirm the discrimination of the different environmental compartments of the sequence soil – phyllosphere – cow-teat - milk and identify the phyla or subphyla discriminating them. This analysis was based either on prokaryotic or fungal community composition of each compartment of the sequence, respectively. A filtered taxonomic matrix was used as the input file. For this, all the sequences were assigned using the SILVA R132 databases of the BIOCOM-PIPE pipeline^58^, then the abundance for the identical lineages were summed to keep only a single lineage. When there was an Unknown taxonomic rank, we added information from the last known rank to facilitate figures visibility and results interpretation. When taxonomic names are identical between different ranks, a letter has been specified after the taxon (i.e. Gemmatimonadetes p = phylum rank, Gemmatimonadetes c = class rank). Briefly, samples from the different compartments are compared by means of class comparison and consistency and effect size estimation in three steps: Kruskall-Wallis test, Wilcoxon’s test, and finally Linear Discriminant Analysis. The LDA score was set at 3. The export2graphlan framework was used to generate the input files to produce the taxonomic cladogram using the GraPhlAn tool^67^.Based on this analysis, results were represented as a taxonomic tree and an effect size histogram. In the taxonomic tree, phyla, subphyla or classes are colored according to the compartment of the sequence in which they are the most represented and point size being proportional to their relative abundance. In the LDA analysis, phylum, subphylum and class taxonomic levels significantly differing in the considered compartment from the others are ranked according to their importance.

For prokaryotic communities (Supplementary Figure S1 and S2), soil housed *Archaea*, *Chloroflexi*, *Acidobacteria*, *Plactomycetes* and *Delta-proteobacteria*, *Rubrobacteria*, *Actinomicrobia* and *Chitinophagales*; while phyllosphere was characterized by *Cyanobacteria*, *Hymenobacteraceae* and *Spirosomaceae* (*Bacteroidetes*). Cow-teat mainly housed *Coriobacteria* and *Clostridia* except *Ruminococcaceae* and milk had higher relative abundance of *Bacilli*, especially *Lactobacillales* and *Bacillales*. On another hand, most of *Alpha*- and *Delta*-*proteobacteria* were shared between soil and phyllosphere, *Bacteroidales* were shared between soil, cow-teat and milk and *Bacillales* between milk and phyllosphere.

For fungal communities (Supplementary Figure S3 and S4), soil housed *Mucoromycota*, *Chytridiomycota*, *Blastocladiomycota* and most *Agaricomycetes* and *Pezizomycetes*. Phyllosphere had higher relative abundance of *Ustilaginomycetes*, *Taphrinomycetes* and *Microascales* but also of most *Tremellomycetes*. Cow-teat mainly housed fewer fungal taxa belonging to *Leotiomycetes*, *Cystobasidiomycetes*, *Microboryomycetes* and *Neocallimastigomycota. M*ilk had particularly high amounts of *Eurotiomycetes* and *Saccharomycetes*. Fungal taxa shared between the different compartments of the sequence were mainly represented by *Ascomycota*.

**Supplementary Figure S1. Taxonomic tree from LefSe analysis for prokaryotic communities along the sequence soil – phyllosphere – cow-teat – milk.** Colors identify the compartment in the sequence: soil: brown; phyllosphere: green; cow-teat: blue; milk: grey. Point size represent the relative abundance of the group in the considered compartment. Codes are reported in the legend and refer to different taxonomic levels from phylum to genus.

**Supplementary Figure S2. LDA diagram from LefSe analysis for prokaryotic communities along the sequence soil – phyllosphere – cow-teat – milk.** Colors identify the compartment in the sequence: soil: brown; phyllosphere: green; cow-teat: blue; milk: grey. Histogram represent length correspond to the LDA score.

**Supplementary Figure S3. Taxonomic tree from LefSe analysis for fungal communities along the sequence soil – phyllosphere – cow-teat – milk.** Colors identify the compartment in the sequence: soil: brown; phyllosphere: green; cow-teat: blue; milk: grey. Point size represent the relative abundance of the group in the considered compartment. Codes are reported in the legend and refer to different taxonomic levels from phylum to genus.

**Supplementary Figure S4. LDA diagram from LefSe analysis for fungal communities along the sequence soil – phyllosphere – cow-teat – milk.** Colors identify the compartment in the sequence: soil: brown; phyllosphere: green; cow-teat: blue; milk: grey. Histogram represent length correspond to the LDA score.

**Supplementary Figure S5. Response of Soil – Phyllosphere correlation coefficient to Total_manure and pH.** The two other explanatory variables of the model (Table 3), *a.k.a* SpRichness and grass, several sets of values are used combining minimum, mean and maximum since the response of the correlation coefficient to these variables is monotonic and positive. Points represent the observed values and response surface represent the predicted values by the model. Colors represent the value of the correlation coefficient.


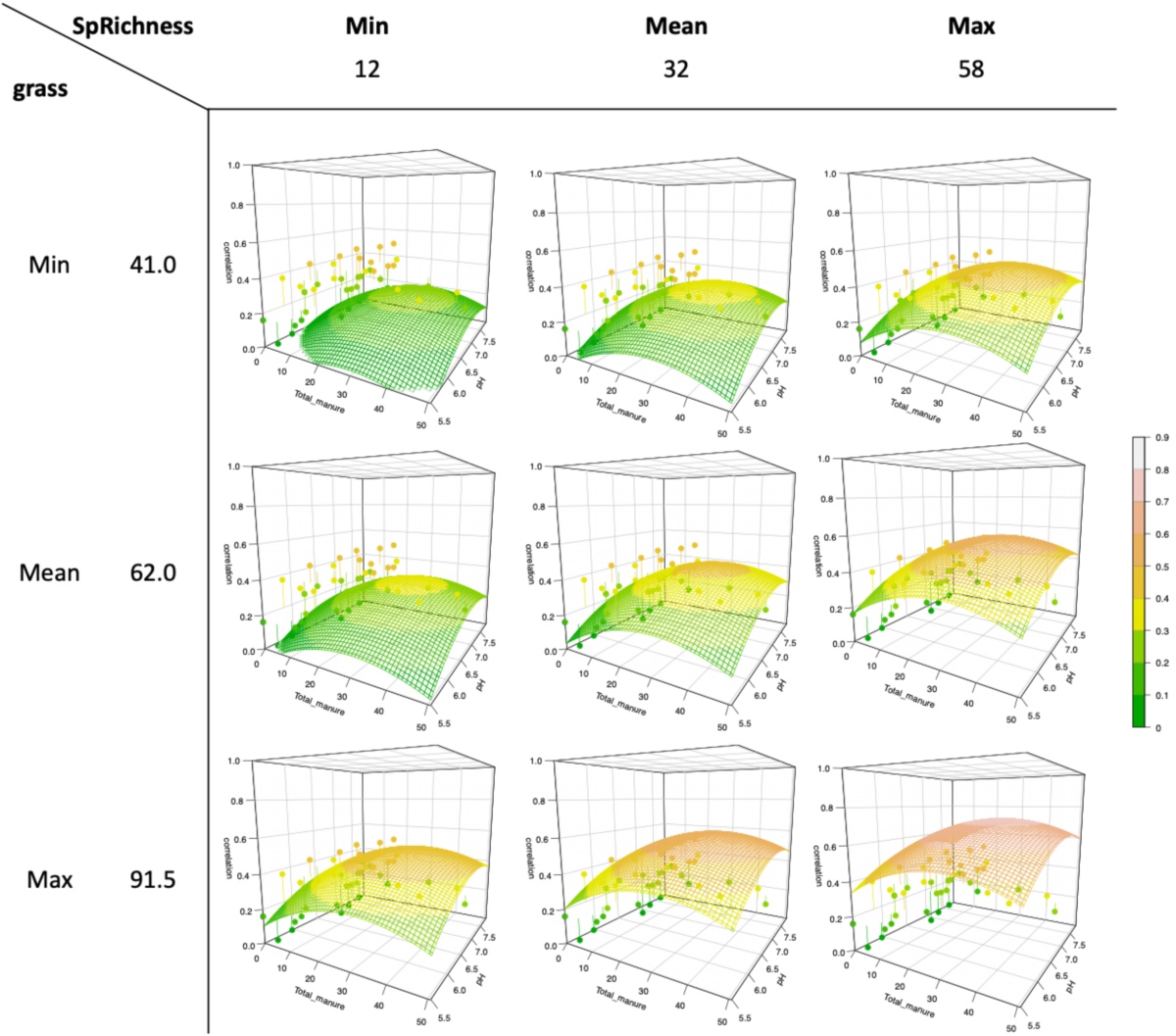


**Supplementary Figure S6. Response of Cow-teat – Milk correlation coefficient to Cattle_spring and Total_manure.** The other explanatory variable of the model (Table 3), a.k.a Cow-teat care, was set to yes or no. Points represent the observed values and response surface represent the predicted values by the model. Colors represent the value of the correlation coefficient.


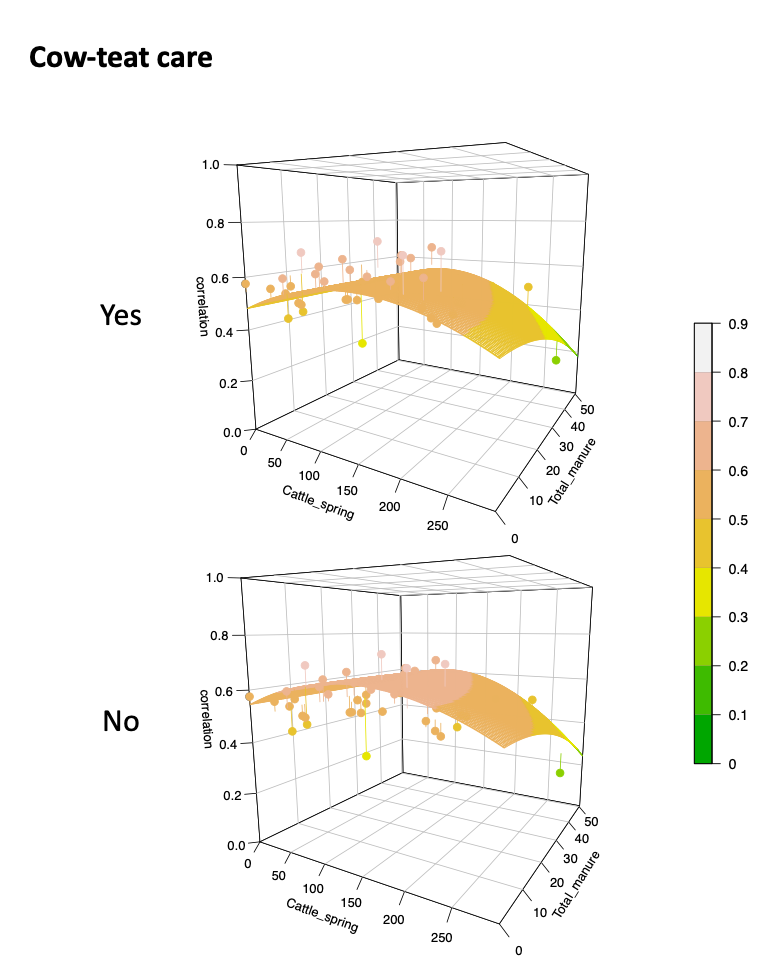

Supplement: Supplementary file 1 — Supplementary Information 1. [file 41598_2021_97373_MOESM1_ESM.docx]
